# Supplementary material for: Interleukin-11-expressing fibroblasts have a unique gene signature correlated with poor prognosis of colorectal cancer
Source: Nat Commun. 2021 Apr 16;12:2281. doi: 10.1038/s41467-021-22450-3 (PMC8052408; doi:10.1038/s41467-021-22450-3)
Supplement: Supplementary file 1 — Supplementary Information [file 41467_2021_22450_MOESM1_ESM.pdf]

Supplementary Information

Interleukin-11-expressing fibroblasts have a unique gene signature correlated with poor prognosis of colorectal cancer

Takashi Nishina[1, *], Yutaka Deguchi[1], Daisuke Ohshima[2], Wakami Takeda[1, 3], Masato Ohtsuka[4, 5], Shigeyuki Shichino[6], Satoshi Ueha[6], Soh Yamazaki[1], Mika Kawauchi[1], Eri Nakamura[7], Chiharu Nishiyama[3], Yuko Kojima[8], Satomi Adachi-Akahane[2], Mizuho Hasegawa[9], Mizuho Nakayama[10], Masanobu Oshima[10], Hideo Yagita[11], Kazutoshi Shibuya[12], Tetuo Mikami[13], Naohiro Inohara[9], Kouji Matsushima[6], Norihiro Tada[7], Hiroyasu Nakano[1, 14, *]

Supplementary Figures 1-10

Supplementary Tables 1-3

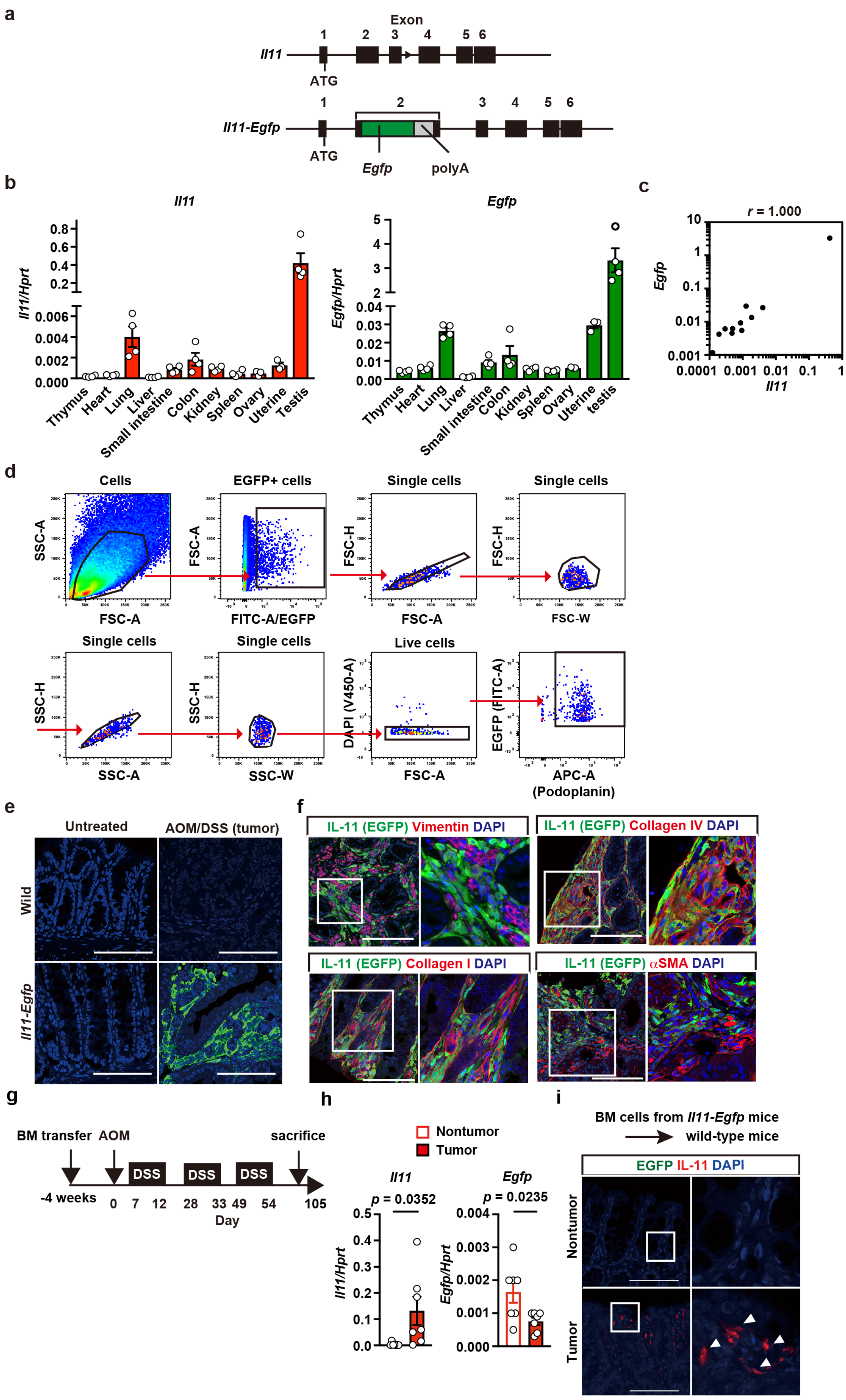
a
Exon
1 2 3 4 5 6
Il11
ATG
Il11-Egfp
Egfp
polyA
b
Il11
Il11/Hprt
Egfp
Egfp/Hprt
Thymus
Heart
Lung
Liver
Small intestine
Colon
Kidney
Spleen
Ovary
Uterine
Testis
c
r = 1.000
Egfp
Il11
d
Cells
EGFP+ cells
Single cells
Live cells
SSC-A
FSC-A
FITC-A/EGFP
FSC-H
FSC-W
SSC-H
SSC-W
DAPI (V450-A)
EGFP (FITC-A)
APC-A
(Podoplanin)
e
Untreated
AOM/DSS (tumor)
Wild
Il11-Egfp
f
IL-11 (EGFP) Vimentin DAPI
IL-11 (EGFP) Collagen IV DAPI
IL-11 (EGFP) Collagen I DAPI
IL-11 (EGFP) αSMA DAPI
g
BM transfer AOM
sacrifice
DSS
-4 weeks 0 7 12 28 33 49 54 105
Day
h
Nontumor
Tumor
Il11
p = 0.0352
Il11/Hprt
Egfp
p = 0.0235
Egfp/Hprt
i
BM cells from Il11-Egfp mice
wild-type mice
EGFP IL-11 DAPI
Nontumor
Tumor

Supplementary Fig. 1. Characterization of IL-11$^+$ cells in CAC using *Il11-Egfp* reporter mice. **a** Diagram of the vector used to construct *Il11-Egfp* reporter mice. The upper panel is the organization of the murine *Il11* gene. An in-frame insertion of an *Egfp-polyA* cassette into the second exon of the *Il11* gene resulted in the expression of EGFP with three additional N-terminal amino acids (lower panel). **b, c** *Il11* and *Egfp* expression in various tissues of *Il11-Egfp* reporter mice. Total RNA was prepared from the indicated mouse tissues from *Il11-Egfp* reporter mice, and *Il11* and *Egfp* expression were determined by qPCR (**b**). Results are mean ± SEM. n = 4 except for ovary or uterine where n = 3 mice. Correlation between *Egfp* and *Il11* expression levels (**c**). *Egfp* and *Il11* expression levels were plotted on the X and Y axis, respectively, and the correlation coefficient (r) and p-value (two-tailed) were calculated using the Pearson correlation test. P = 0.0001. **d** Gating strategy to analyze EGFP$^+$ cells. **e, f** WT and *Il11-Egfp* mice were untreated or treated with AOM/DSS as in Fig. 1a; colon tissue (**e**, left panels) or tumor tissue sections (**e**, right panels and **f**) were immunostained with anti-GFP (**e**) (n = 2 mice) or the indicated antibodies (**f**). n = 4 (upper panels) and 3 (lower panels) mice. Results are merged images, and the right panels are enlarged images of the boxes. Scale bar, 100 µm. **g** Protocol for induction of AOM/DSS-induced CAC in BM-transferred mice. BM cells from 8-week-old *Il11-Egfp* mice were transferred to lethally irradiated 4- to 5-week-old wild-type mice. One month following reconstitution, mice were treated with AOM/DSS as in Fig. 1a. **h, i** Expression of *Egfp* is not elevated in tumor tissues of BM-transferred mice. Tumor and non-tumor tissues were removed from BM-transferred mice after treatment with AOM/DSS, and *Il11* and *Egfp* expression levels were determined using qPCR (**h**). Results are mean ± SEM (n = 7 mice). Pooled data from two independent experiments. Statistical significance was determined using the two-tailed unpaired Student's t-test. *$P < 0.05$. Tumor tissue sections were stained with anti-IL-11 and anti-GFP antibodies (**i**). Results are merged images, and the right panels are enlarged images of the boxes (n = 4 mice). White arrowheads indicate IL-11$^+$ EGFP$^-$ cells. Scale bar, 100 µm. Source data are provided as a Source Data file.

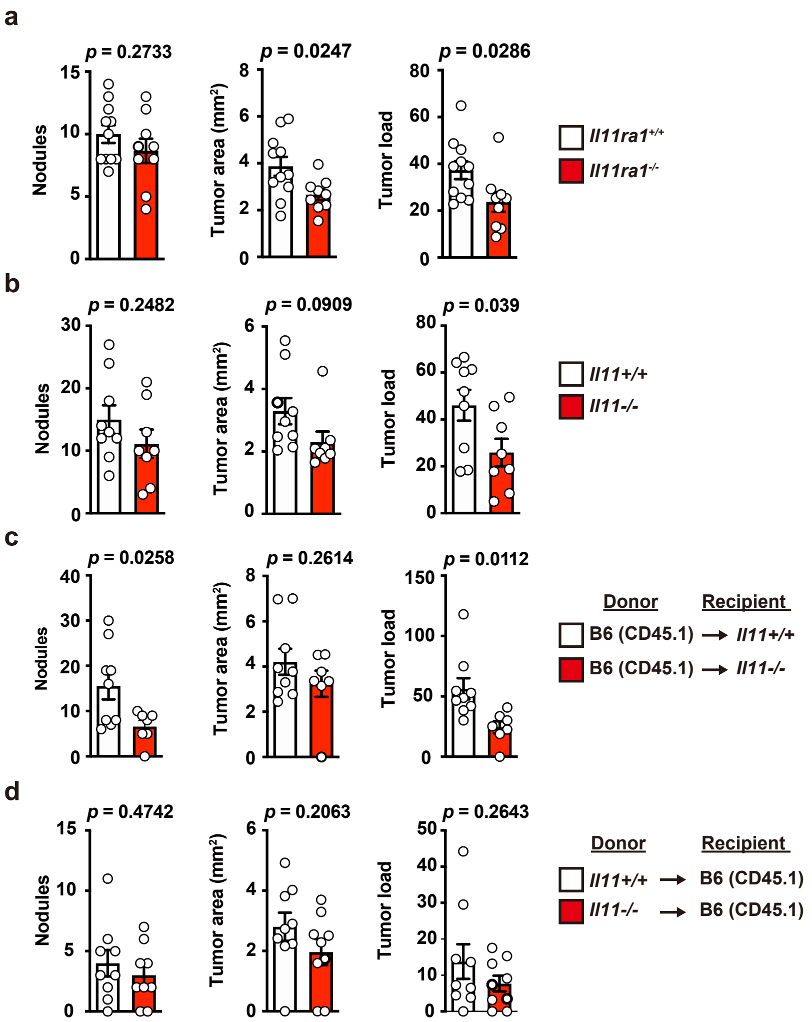

Supplementary Fig. 2. Attenuated CAC development in *Il11ra1*$^{-/-}$ and *Il11*$^{-/-}$ mice. **a** *Il11ra1*$^{+/+}$ and *Il11ra1*$^{-/-}$ mice were treated as in Fig. 1a. On day 84 to 98 after AOM/DSS treatment, mice were sacrificed, and tumor numbers and areas were determined. Tumor load was calculated as described in the methods, and tumor numbers, average tumor area, and the tumor load of an individual mouse are shown. Results are mean ± SEM. n = 11 (*Il11ra1*$^{+/+}$) or 9 (*Il11ra1*$^{-/-}$) mice; pooled data from four independent experiment. **b** *Il11*$^{+/+}$ and *Il11*$^{-/-}$ mice were treated as in Fig. 1a and analyzed as in Supplementary Fig. 2a. Results are mean ± SEM. n = 9 (*Il11*$^{+/+}$) or 8 (*Il11*$^{-/-}$) mice; pooled data from two independent experiment. **c, d** Deletion of *Il11* in non-hematopoietic cells attenuates the development of CAC in mice. BM cells from mice of the indicated genotypes were prepared and transferred to the indicated mice that had been lethally irradiated. At 2 months after BM transfer, mice were treated as in Fig. 1a and analyzed as in Supplementary Fig. 2a. Results are mean ± SEM (**c**). n = 9 (B6 to *Il11*$^{+/+}$) or 7 (B6 to *Il11*$^{-/-}$) mice; pooled data from two independent experiment. **d**, n = 9 (*Il11*$^{+/+}$ to B6) or 9 (*Il11*$^{-/-}$ to B6) mice; pooled data from three independent experiment. Statistical significance

was determined using the two-tailed unpaired Student's *t*-test (**a**–**d**). Source data are provided as a Source Data file.

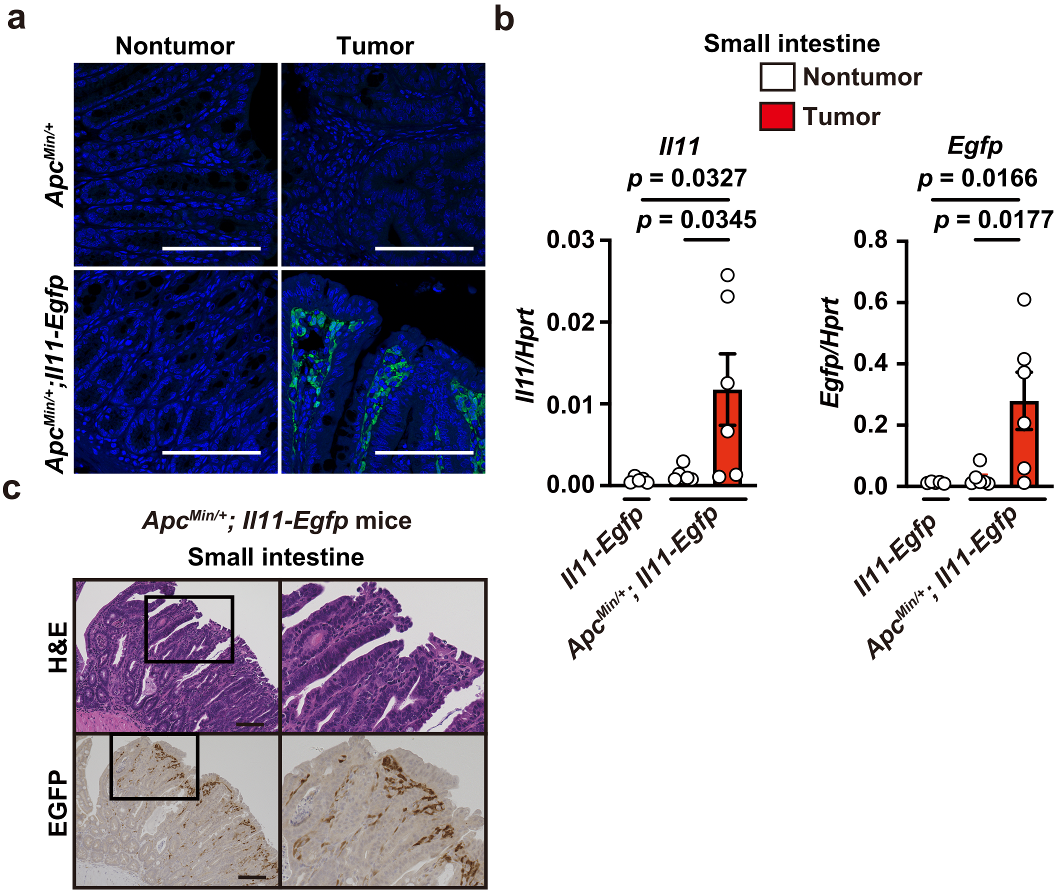

Supplementary Fig. 3. IL-11^{+} cells also appear in tumor tissues in the small intestine of *Apc$^{Min/+}$;Il11-Egfp* reporter mice. **a** Colon sections from *Apc$^{Min/+}$* mice and *Apc$^{Min/+}$;Il11-Egfp* reporter mice were stained with anti-GFP antibody (n = 2). Scale bar, 100 µm. **b** *Il11* and *Egfp* expression is elevated in tumors, but not in nontumor tissues in the small intestine from 20- to 24-week-old *Apc$^{Min/+}$;Il11-Egfp* reporter mice, or in normal small intestine from *Il11-Egfp* reporter mice. *Il11* and *Egfp* mRNA expression levels were determined using qPCR. Results are mean ± SEM. *n* = 5 (*Il11-Egfp*) or 6 (nontumor or tumor of *Apc$^{Min/+}$; Il11-Egfp*) mice. Pooled results from two independent experiments. Statistical significance was determined using the one-way ANOVA with Tukey's multiple comparison. **c** Tumor sections in the small intestine from *Apc$^{Min/+}$;Il11-Egfp* reporter mice were stained with H&E (upper panels) or anti-GFP antibody (lower panels) (*n* = 6 mice). The right panels show enlargements of the black boxes from the left panels. Scale bar, 100 µm. Source data are provided as a Source Data file.

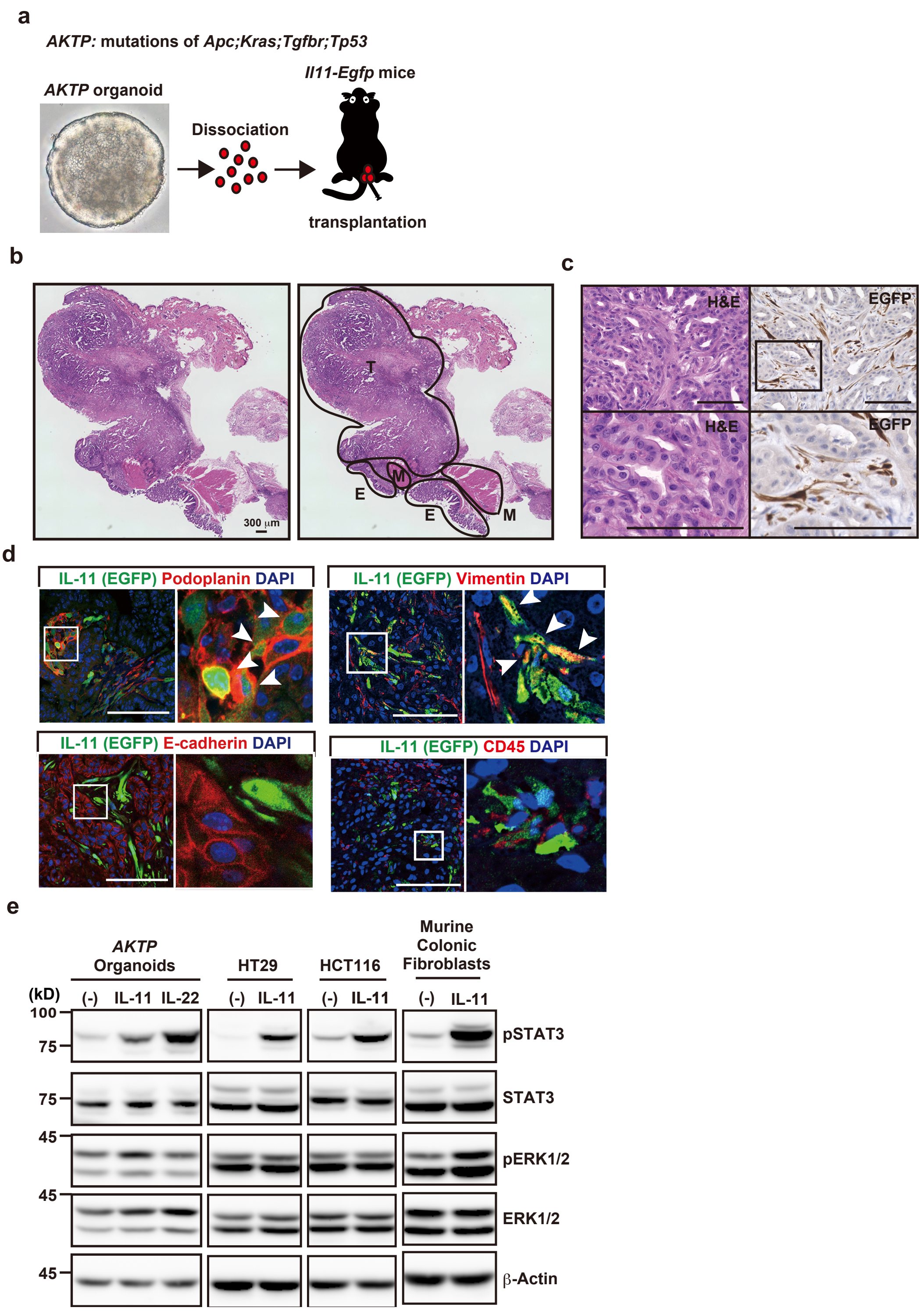

Supplementary Fig. 4. Transplantation of tumor organoids induces IL-11$^+$ fibroblasts. **a** Schema of transplantation of the tumor organoids derived from *AKTP* mice. The tumor organoids from *AKTP* mice were expanded using as a standard procedure. After dissociation, cells were transplanted into the rectums of *Il11-Egfp* reporter mice. **b, c** Tumor organoids developed large tumors, and IL-11$^+$ fibroblasts appeared in the tumors.

A representative of the whole tumor image with adjacent normal tissues in the rectum (**b**). E, M, and T indicate normal epithelial cells, muscles, and tumor tissues, respectively. Colon tumor sections were stained with H&E or anti-GFP antibody (**c**). Scale bar, 100 μm. **d** Tumor sections were stained with the indicated antibodies (n = 4). White arrowheads indicate $EGFP^+$ cells expressing podoplanin or vimentin. Scale bar, 100 μm. **e** Colon tumor organoids derived from *AKTP* mice, human colon cancer cell lines (HT29 and HCT116), and colon fibroblasts from wild-type mice were untreated or stimulated with 100 ng/mL of murine IL-11, murine IL-22, or human IL-11. Cell lysates were analyzed by immunoblotting with the indicated antibodies. Results are representative of two independent experiments.

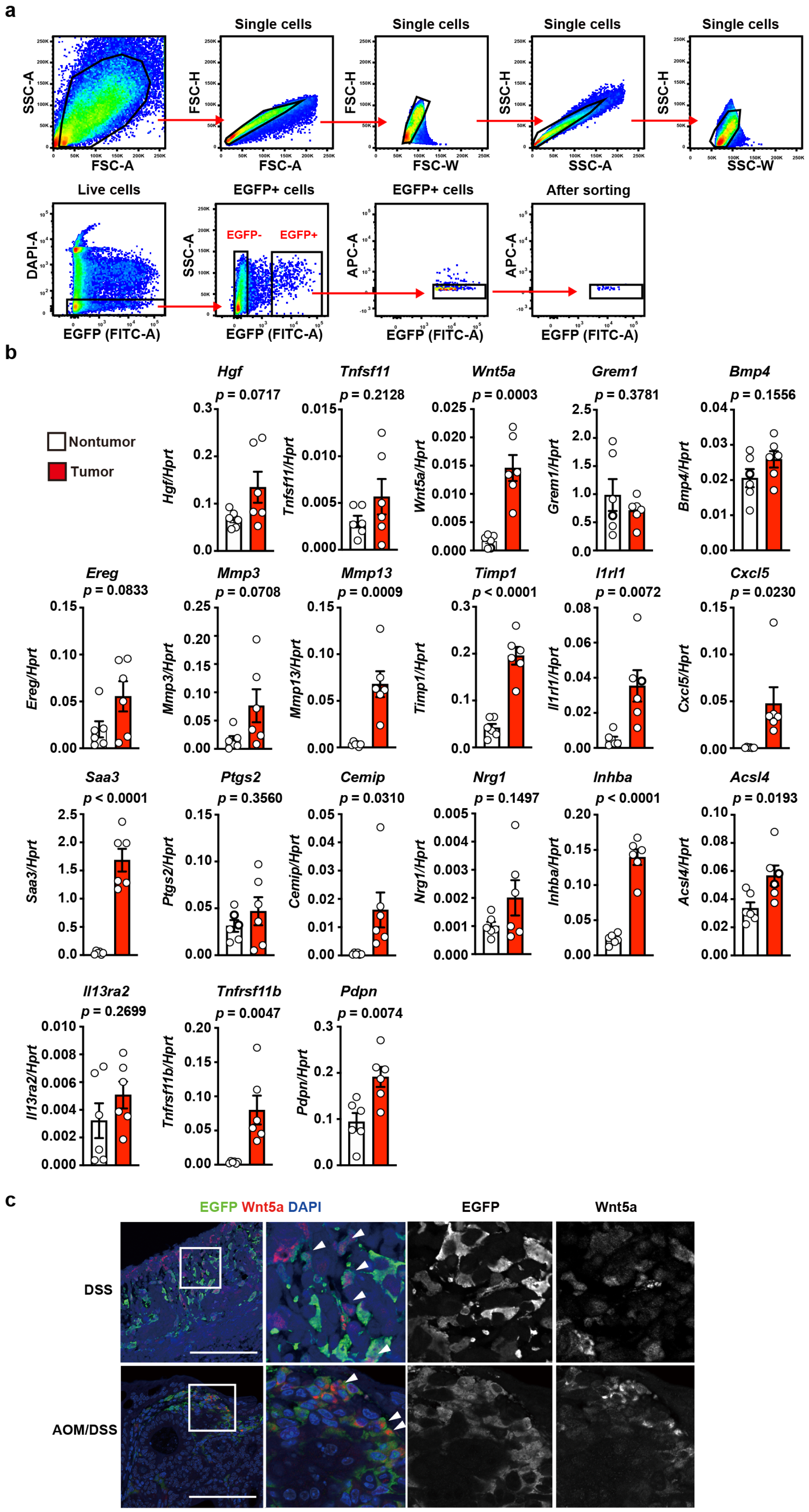
a
Single cells
Single cells
Single cells
Single cells
SSC-A
FSC-A
FSC-H
FSC-A
FSC-H
FSC-W
SSC-H
SSC-A
SSC-H
SSC-W
Live cells
EGFP+ cells
EGFP+ cells
After sorting
DAPI-A
EGFP (FITC-A)
SSC-A
EGFP-
EGFP+
EGFP (FITC-A)
APC-A
EGFP (FITC-A)
APC-A
EGFP (FITC-A)
b
Nontumor
Tumor
Hgf p = 0.0717
Tnfsf11 p = 0.2128
Wnt5a p = 0.0003
Grem1 p = 0.3781
Bmp4 p = 0.1556
Ereg p = 0.0833
Mmp3 p = 0.0708
Mmp13 p = 0.0009
Timp1 p < 0.0001
Il1rl1 p = 0.0072
Cxcl5 p = 0.0230
Saa3 p < 0.0001
Ptgs2 p = 0.3560
Cemip p = 0.0310
Nrg1 p = 0.1497
Inhba p < 0.0001
Acsl4 p = 0.0193
Il13ra2 p = 0.2699
Tnfrsf11b p = 0.0047
Pdpn p = 0.0074
Hgf/Hprt
Tnfsf11/Hprt
Wnt5a/Hprt
Grem1/Hprt
Bmp4/Hprt
Ereg/Hprt
Mmp3/Hprt
Mmp13/Hprt
Timp1/Hprt
Il1rl1/Hprt
Cxcl5/Hprt
Saa3/Hprt
Ptgs2/Hprt
Cemip/Hprt
Nrg1/Hprt
Inhba/Hprt
Acsl4/Hprt
Il13ra2/Hprt
Tnfrsf11b/Hprt
Pdpn/Hprt
c
EGFP Wnt5a DAPI
EGFP
Wnt5a
DSS
AOM/DSS

Supplementary Fig. 5. The expression of genes enriched in IL-11$^+$ fibroblasts is elevated in colon tumor tissues of mice. **a** Sorting strategy to isolate EGFP$^+$ cells from the colon of *Il11-Egfp* reporter mice. *Il11-Egfp* reporter mice were treated as in Fig.4. Ter119$^+$ CD45$^+$ CD31$^+$ EpCAM$^+$ cell populations were depleted using MojoSort Mouse anti-APC Nanobeads (BioLegend) and MojoSort Magnet (BioLegend), and then sorted by BD FACSAriaTM III Cell Sorter (BD Biosciences). **b** *Il11-Egfp* reporter mice were treated as in Fig.1a. mRNAs were prepared from tumors and nontumor colon tissue of mice on day 98−105 after AOM/DSS treatment, and the expression levels of the indicated genes were determined using qPCR. Results are mean ± SEM (n = 6 mice). Representative results of two independent experiments. Statistical significance was determined using the two-tailed unpaired Student's t-test. **c** IL-11$^+$ fibroblasts co-express Wnt5a. *Il11-Egfp* reporter mice were treated with DSS or AOM/DSS as described in Figs. 1a and 3a. Colon tissue (upper panels) or colon tumor sections (lower panels) were stained with the indicated antibodies (n = 3 mice). Right-hand panels show enlarged images of the white boxes on the left. White arrowheads indicate IL-11$^+$ fibroblasts expressing Wnt5a. Scale bar, 100 μm. Source data are provided as a Source Data file.

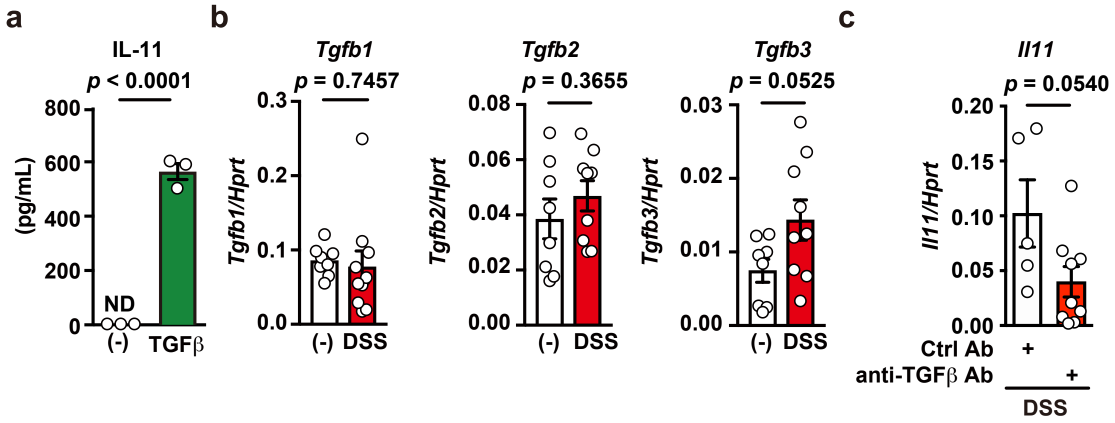

Supplementary Fig. 6. TGFβ is not involved in the upregulation of colon *Il11* expression in DSS-treated mice. **a** TGFβ induces IL-11 production by colon fibroblasts. Colon fibroblasts were prepared from wild-type mice and left untreated or stimulated with TGFβ (100 ng/mL) for 16 hours. Concentrations of TGFβ in the culture supernatant were determined using ELISA. Results are mean ± SD of triplicate samples and representative of two independent experiments. **b** WT mice were treated with 1.5% DSS as in Fig. 3a. Expression of *Tgfb*1-3 in the colon of mice on day 5 after DSS treatment was determined using qPCR. Results are mean ± SEM. n = 8 (untreated), 10 (*Tgfb1*, DSS), or 9 (*Tgfb2* and *Tgfb3*, DSS) mice. Pooled data from two independent experiments. **c** WT mice were intraperitoneally administered 100 μg control IgGs or anti-TGFβ antibody on days 2 and 4 after DSS administration and sacrificed on day 5. Expression of *Il11* in the colon was determined using qPCR. Results are mean ± SE. n = 5 (control Ab) or 9 (anti-TGFβ Ab) mice. Pooled data from two independent experiments. Statistical significance was determined using the unpaired two-tailed Student's t-test (**a, b**) or two tailed Whitney U test (**c**). ND, not detected. Source data are provided as a Source Data file.

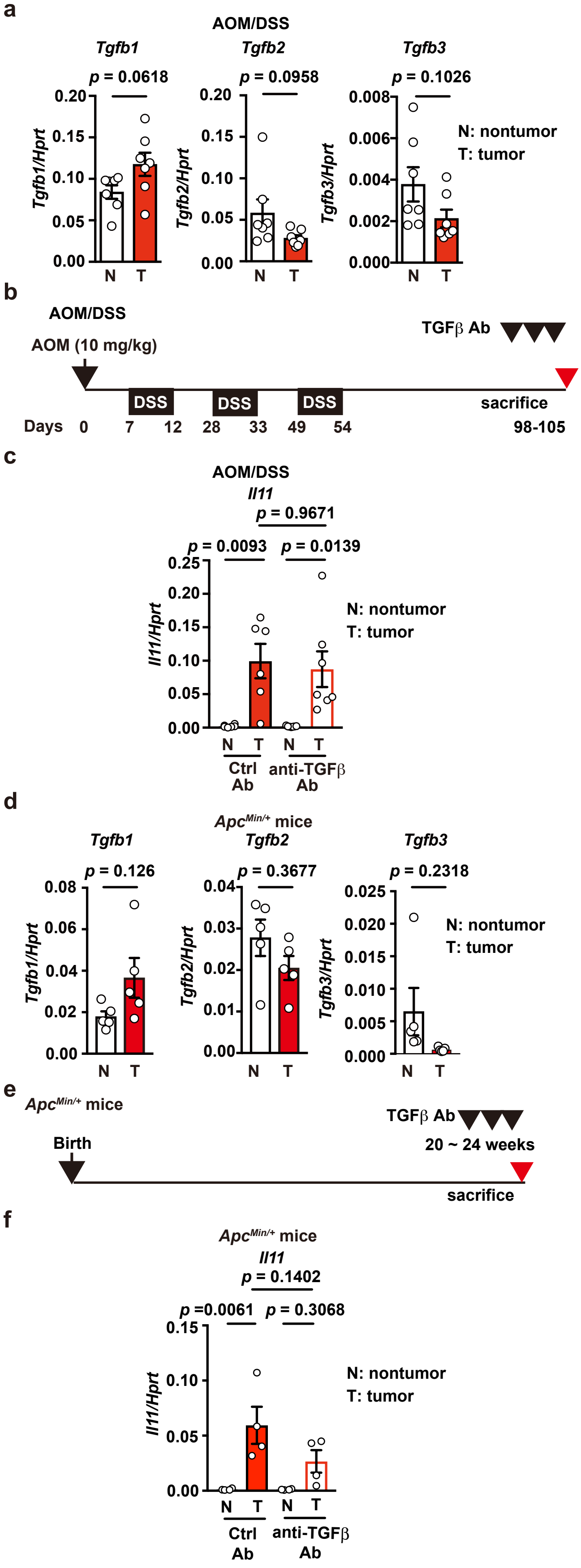

a
AOM/DSS
Tgfb1
Tgfb2
Tgfb3
p = 0.0618
p = 0.0958
p = 0.1026
Tgfb1/Hprt
Tgfb2/Hprt
Tgfb3/Hprt
N: nontumor
T: tumor
b
AOM/DSS
AOM (10 mg/kg)
TGFβ Ab
DSS
sacrifice
Days 0 7 12 28 33 49 54 98-105
c
AOM/DSS
Il11
p = 0.9671
p = 0.0093
p = 0.0139
Il11/Hprt
N: nontumor
T: tumor
Ctrl Ab
anti-TGFβ Ab
d
ApcMin/+ mice
Tgfb1
Tgfb2
Tgfb3
p = 0.126
p = 0.3677
p = 0.2318
Tgfb1/Hprt
Tgfb2/Hprt
Tgfb3/Hprt
N: nontumor
T: tumor
e
ApcMin/+ mice
Birth
TGFβ Ab
20 ~ 24 weeks
sacrifice
f
ApcMin/+ mice
Il11
p = 0.1402
p =0.0061
p = 0.3068
Il11/Hprt
N: nontumor
T: tumor
Ctrl Ab
anti-TGFβ Ab

Supplementary Fig. 7. TGFβ is not involved in the upregulation of colon *Il11* expression in AOM/DSS-treated mice and *Apc*$^{Min/+}$ mice. **a** *Tgfb1–3* expression in tumors and nontumor tissues in colon from AOM/DSS-treated mice. On day 98–105 after AOM injection, mRNA was extracted from tumor (T) and non-tumor tissues (N), and the expression levels of *Tgfb*1–3 were determined using qPCR. Results are mean ± SEM (n = 7 mice). Results are representative of two independent experiments. **b, c** After induction of colorectal tumors after AOM/DSS treatment, mice were treated with control IgGs or anti-TGFβ antibody on days −1, −3, and −5 (before sacrifice) (**b**). *Il11* expression was determined using qPCR (**c**). Results are mean ± SEM. n = 6 (control Ab) or 7 (anti-TGFβ Ab) mice; pooled data from two independent experiments. **d** *Tgfb1–3* expression levels in tumors and nontumor tissues in colon from *Apc*$^{min/+}$ mice were determined using qPCR. Results are mean ± SEM (n = 5 mice). Results are representative of two independent experiments. **e, f** *Apc*$^{min/+}$ mice were given intraperitoneal injections of control mouse IgGs or anti-TGFβ antibody (days −1, −3, −5) before sacrifice (**e**). *Il11* expression in tumors and nontumor tissues was determined using qPCR (**f**). Results are mean ± SE (n = 4 mice). Pooled data from three independent experiments. Statistical significance was determined using the two-tailed unpaired Student's *t*-test (**a, d**) or two-way ANOVA with Tukey's multiple comparison test (**c, f**). Source data are provided as a Source Data file.

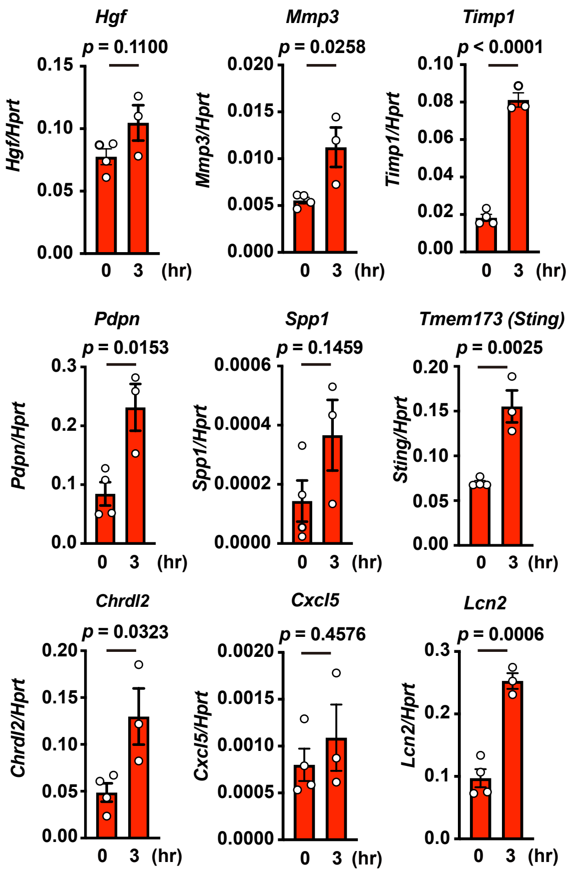

Supplementary Fig. 8. Administration of an IL-11R agonist induces expression of a set of genes enriched in IL-11^{+} fibroblasts in whole colon in wild-type mice. **a** Wild-type mice were treated with an IL-11R agonist as in Fig. 6c for 3 hours. Expression of the indicated genes of the whole colon was determined using qPCR. Results are mean ± SEM. n = 4 (untreated) or 3 (IL-11R agonist-treated) mice. Statistical significance was determined using the two-tailed unpaired Student's t-test. Results are representative of two independent experiments. Source data are provided as a Source Data file.

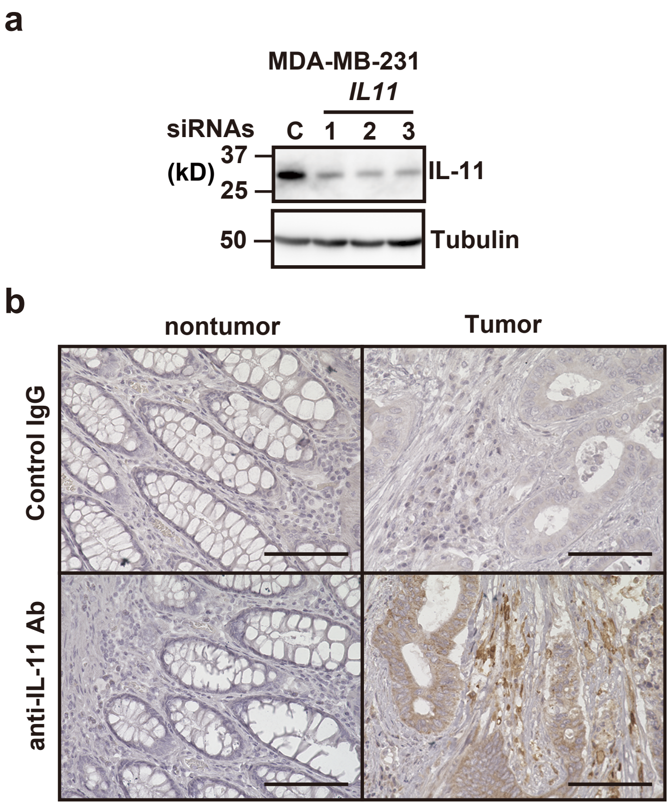

Supplementary Fig. 9. The specificity of anti-IL-11 antibody used in this study. **a** Verification of anti-IL-11 antibody used in this study by Western blotting. MDA-MB-231 cells were treated with control (C) or three different siRNAs against human *Il11*, and IL-11 expression in cell lysates was analyzed by Western blotting with anti-IL-11 and anti-tubulin antibodies. Results are representative of two independent experiments. **b** Tissues of nontumor and advanced colorectal cancers were stained with anti-IL-11 antibody or control rabbit IgG (n = 2 human samples). Scale bar, 100 μm.

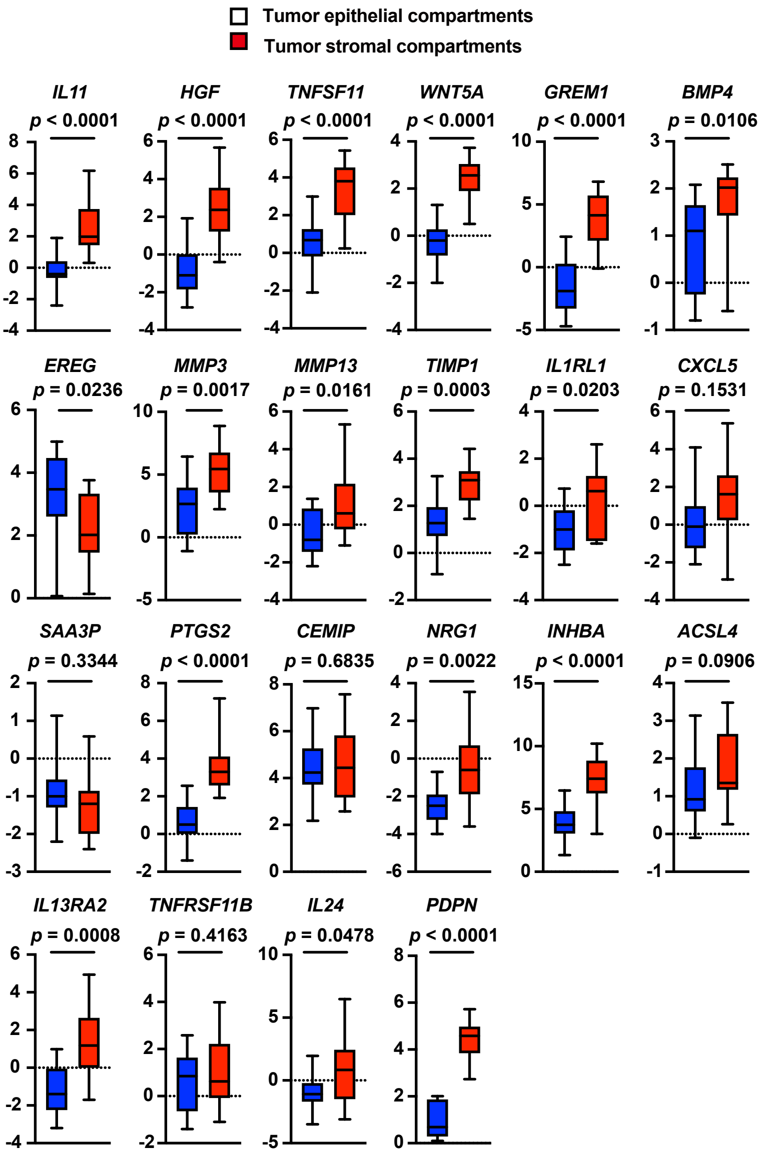

Supplementary Fig. 10. The expression of some enriched genes in IL-11^{+} fibroblasts is elevated in human tumor stromal compartments compared with tumor epithelial compartments. From the data set (GSE35602) that includes gene expression data of epithelium and stroma isolated from normal and colon cancer tissues using laser-micro dissection, we retrieved the expression levels of the genes enriched in IL-11^{+} fibroblasts. The LOWESS normalized signaling intensities of each gene in tumor stromal compartments and tumor epithelial compartments are normalized with the average of normal epithelial compartment intensities. Average signaling intensities of non-tumor epithelial compartments were adjusted to be 0. Normalized intensities of each gene are shown by box-and-whisker plots. Boxes and whiskers show the 25th–75th percentile with the median and the minimum–maximum, respectively (n = 13).

Statistical significance was determined using the two-tailed unpaired Student's *t*-test. Source data are provided as a Source Data file.

Supplementary Table 1. Clinical information of human colorectal tumor samples.

	Adenoma	Early	Advanced
Age (years)	67.9 ± 9.5	69.5 ± 10.9	68.1 ± 8.4
Sex	Male 9, female 2	Male 7, female 3	Male 8, female 2
Stages	Adenoma	T1b (10)	T1 (1), T3 (6), T4 (3)
Differentiation		Moderate ~ highly differentiated	Moderate ~ highly differentiated

Supplementary Table 2. Genotypes, ages, and genders of mice used in the experiments.

Figures		Treatment	Genotypes	Ages	Sex
Figure 1	a-l	AOM/DSS	Il11-Egfp mice	9-15-week-old	female
Figure 2	a-g	untreated	ApcMin/+; Il11-Egfp mice	20-24-week-old	female and male
	I, j	untreated	ApcMin/+; Il11+/- mice	20-24-week-old	female and male
			ApcMin/+; Il11-/- mice	20-24-week-old	female and male
Figure 3	a	DSS	B6 mice	12-15-week-old	male
	b	DSS	Il11-Egfp mice	12-13-week-old	male
Figure 4		DSS	Il11-Egfp mice	13-15-week-old	male
Figure 5	a	DSS + NAC	B6 mice	10-12-week-old	male
	b	DSS + Abx	B6 mice	10-12-week-old	male
	c	DSS + NAC	B6 mice	10-12-week-old	male
	d	DSS + Abx	B6 mice	12-14-week-old	male
	e	DSS + Trametinib	B6 mice	8-10-week old	male
Figure 6		AOM/DSS	B6 mice	14-15-week-old	female and male
Figure 7b		NAC, Abx, Trametinib	ApcMin/+ mice	20-24-week-old	female and male
Supple. Fig. 1	b	untreated	Il11-Egfp mice	8-15 week-old	female and male
	e-f	untreated, AOM/DSS	B6, Il11-Egfp mice	9-15-week-old	female
	h	AOM/DSS/BM transfer	B6 mice	4-5-week-old	female
Supple. Fig. 2		AOM/DSS	Il11ra1+/+, Il11ra1-/- mice	9-15-week-old	female
			Il11+/+, Il11-/- mice	9-15 week-old	female
			WT BM to Il11+/+ or Il11-/- mice	6-week-old	female
			Il11+/+ or Il11-/- BM to B6 mice	6-week-old	female
Supple. Fig. 3	b	untreated	ApcMin/+, ApcMin/+; Il11-Egfp	20-24-week-old	female and male
Supple. Fig. 4	a	Tumor implantation	Il11-Egfp mice	7-9-week-old	male
Supple. Fig. 5		AOM/DSS	Il11-Egfp mice	15-16-week-old	female
Supple. Fig. 6	b	DSS	B6 mice	10-12-week-old	male
	c	DSS + TGFb Ab	B6 mice	11-14-week-old	male
Supple. Fig. 7	a	AOM/DSS	Il11-Egfp mice	15-16-week-old	female
	c	AOM/DSS + TGFb	Il11-Egfp mice	15-16-week-old	female
	d	untreated	ApcMin/+ mice	20-24-week-old	female and male
	f	anti-TGFb	ApcMin/+ mice	20-24-week-old	female and male
Supple. Fig. 8		IL11-R agonist	B6 mice	8-week-old	female

Supplementary Table 3. Primer list used in this study.

Acsl4	5’ - CTTCCTCTTAAGGCCGGGAC - 3’
	5’ - TGCCATAGCGTTTTTCTTAGATTT - 3’
Bmp4:	5’ - TTCCTGGTAACCGAATGCTGA - 3’
	5’ - CCTGAATCTCGGCGACTTTTT - 3’
Cemip:	5’- CTCCTGGCCAACTTCTCAGG - 3’
	5’- TGCCATGGCCAATGTGTACT - 3’
Chrdl2:	5’ - TTTGCTGGGACTCGTGATGTT - 3’
	5’ - GTGGTTCCAAGTAGGGGTGC - 3’
Cxcl5:	5’ - GTTCCATCTCGCCATTCATGC - 3’
	5’ - GCGGCTATGACTGAGGAAGG - 3’
Ereg:	5’ - TGCCTCTTGGGTCTTGACG - 3’
	5’ - ACTTTGTAATCTGCACTTGAGCC - 3’
Grem1:	5’ - TCAAAGCGGGCACATTCAG - 3’
	5’ - AGTAGGAATCGGGTGGTTTGG - 3’
Egfp:	5’ - AGCAAAGACCCCAACGAGAA - 3’
	5’ - GGCGGCGGTCACGAA - 3’
Hgf:	5’ - ATGTGGGGGACCAAACTTCTG - 3’
	5’ - GGATGGCGACATGAAGCAG - 3’
Hmox1:	5’ - GTCAAGCACAGGGTGACAGA - 3’
	5’- ATCACCTGCAGCTCCTCAAA - 3’
Hprt:	5’ - AACAAAGTCTGGCCTGTATCCAA - 3’
	5’ - GCAGTACAGCCCCAAAATGG - 3’
Il6st:	5’ - CCGTGTGGTTACATCTACCCT - 3’
	5’ - CGTGGTTCTGTTGATGACAGTG - 3’

Il11:	5’ - CTGCACAGATGAGAGACAAATTCC - 3’
	5’ - GAAGCTGCAAAGATCCCAATG - 3’
Il11ra:	5’ - AACAGATGCTGTGGCTGGG - 3’
	5’ - CAGGGGACCAGTGCTAGGAG - 3’
Il22ra:	5’ - ATGAAGACACTACTGACCATCCT - 3’
	5’ - CAGCCACTTTCTCTCTCCGT - 3’
Inhba:	5’ - TGAGAGGATTTCTGTTGGCAAG - 3’
	5’ - TGACATCGGGTCTCTTCTTCA - 3’
Il1rl1:	5’ - TGACACCTTACAAAACCCGGA - 3’
	5’ - AGGTCTCTCCCATAAATGCACA - 3’
Il13ra2:	5’ - ACCGAAATGTTGATAGCGACAG - 3’
	5’ - ACAATGCTCTGACAAATGCGTA - 3’
Lcn2:	5’ - AAGGAGCTGTCCCCTGAACT - 3’
	5’ - GGTGGGGACAGAGAAGATGA - 3’
Mmp3:	5’ - ACATGGAGACTTTGTCCCTTTTG - 3’
	5’ - TTGGCTGAGTGGTAGAGTCCC - 3’
Mmp13:	5’ - CTTCTTCTTGTTGAGCTGGACTC - 3’
	5’ - CTGTGGAGGTCACTGTAGACT - 3’
Nrg1:	5’ - ATGGAGATTTATCCCCCAGACA - 3’
	5’ - GTTGAGGCACCCTCTGAGAC - 3’
Pdpn:	5’ - ACCGTGCCAGTGTTGTTCTG - 3’
	5’ - AGCACCTGTGGTTGTTATTTGT - 3’
Ptgs2:	5’ - TTCAACACACTCTATCACTGGC - 3’
	5’ - AGAAGCGTTTGCGGTACTCAT - 3’

Saa3:	5’ - TGCCATCATTCTTTGCATCTTGA - 3’
	5’ - CCGTGAACTTCTGAACAGCCT - 3’
Spp1:	5’ - CACTCCAATCGTCCCTAC - 3’
	5’ - AGACTCACCGCTCTTCAT - 3’
Tgfb1:	5’ - TTGCTTCAGCTCCACAGAGA - 3’
	5’ - TGGTTGTAGAGGGCAAGGAC - 3’
Tgfb2:	5’ - CTTCGACGTGACAGACGCT - 3’
	5’ - GCAGGGGCAGTGTAAACTTATT - 3’
Tgfb3:	5’ - CAGGCCAGGGCAGTCAGAG - 3’
	5’ - ATTTCCAGCCTAGATCCTGCC - 3’
Timp1:	5’- GCAACTCGGACCTGGTCATAA-3’
	5’- CGGCCCGTGATGAGAAACT-3’
Tmem173:	5’ - GGTCACCGCTCCAAATATGTAG - 3’
	5’ - CAGTAGTCCAAGTTCGTGCGA - 3’
Tnfsf11:	5’ - CAGCATCGCTCTGTTCCTGTA - 3’
	5’ - CTGCGTTTTCATGGAGTCTCA - 3’
Tnfrsf11b:	5’ - ACCCAGAAACTGGTCATCAGC - 3’
	5’ - CTGCAATACACACACTCATCACT - 3’
Wnt5a:	5’ - CAACTGGCAGGACTTTCTCAA - 3’
	5’ - CATCTCCGATGCCGGAACT - 3’
16S rRNA:	5’ - GGTGAATACGTTCCCGG - 3’
	5’ - TACGGCTACCTTGTTACGACTT - 3’
