# Supplementary material for: Interleukin-11-expressing fibroblasts have a unique gene signature correlated with poor prognosis of colorectal cancer
Source: Nat Commun. 2021 Apr 16;12:2281. doi: 10.1038/s41467-021-22450-3 (PMC8052408; doi:10.1038/s41467-021-22450-3)
Supplement: Supplementary file 3 — Description of Additional Supplementary Files [file 41467_2021_22450_MOESM3_ESM.docx]

Description of Additional Supplementary File

1. Title: Supplementary data 1

Description: Genes upregulated and downregulated in cluster 6 patients compared to those in cluster 5 patients.
