# Supplementary material for: Interleukin-11-expressing fibroblasts have a unique gene signature correlated with poor prognosis of colorectal cancer
Source: Nat Commun. 2021 Apr 16;12:2281. doi: 10.1038/s41467-021-22450-3 (PMC8052408; doi:10.1038/s41467-021-22450-3)
Supplement: Supplementary file 5 — Reporting Summary [file 41467_2021_22450_MOESM5_ESM.pdf]

nature research

Corresponding author(s): Takashi Nishina and Hiroyasu Nakano

Last updated by author(s): Mar 25, 2021

Reporting Summary

Nature Research wishes to improve the reproducibility of the work that we publish. This form provides structure for consistency and transparency in reporting. For further information on Nature Research policies, see Authors & Referees and the Editorial Policy Checklist.

Statistics

For all statistical analyses, confirm that the following items are present in the figure legend, table legend, main text, or Methods section.

n/a	Confirmed	
☐	☒	The exact sample size (*n*) for each experimental group/condition, given as a discrete number and unit of measurement
☐	☒	A statement on whether measurements were taken from distinct samples or whether the same sample was measured repeatedly
☐	☒	The statistical test(s) used AND whether they are one- or two-sided<br>*Only common tests should be described solely by name; describe more complex techniques in the Methods section.*
☐	☒	A description of all covariates tested
☐	☒	A description of any assumptions or corrections, such as tests of normality and adjustment for multiple comparisons
☐	☒	A full description of the statistical parameters including central tendency (e.g. means) or other basic estimates (e.g. regression coefficient) AND variation (e.g. standard deviation) or associated estimates of uncertainty (e.g. confidence intervals)
☐	☒	For null hypothesis testing, the test statistic (e.g. *F*, *t*, *r*) with confidence intervals, effect sizes, degrees of freedom and *P* value noted<br>*Give P values as exact values whenever suitable.*
☒	☐	For Bayesian analysis, information on the choice of priors and Markov chain Monte Carlo settings
☒	☐	For hierarchical and complex designs, identification of the appropriate level for tests and full reporting of outcomes
☐	☒	Estimates of effect sizes (e.g. Cohen's *d*, Pearson's *r*), indicating how they were calculated

Our web collection on statistics for biologists contains articles on many of the points above.

Software and code

Policy information about availability of computer code

Data collection

1) all-in-one microscope (BZ-X700, Keyence), BX-63 (Olympus), LSM 880 (Zeiss) and A1R (Nikon) - used to obtain images of mouse tissues
2) Amersham Biosciences imager 600 (GE Healthcare) - used for Western blotting analysis
3) 7500 Real-Time PCR detection system (Applied Biosystems) - used to Quantitative PCR (qPCR) Assays
4) Ion Genestudio S5 Sequencer (Thermo Fisher Scientific) - used to RNA-seq analysis

Data analysis

1) Fiji (ImageJ 2.0.0, https://Fiji.sc) - used for quantification of tumor size in images, quantification of cell numbers and tissue area in images and western blotting data analysis.
2) 7500 SDS software 2.3 (Thermo Fisher Scientific) - used to qPCR Assays
3) FloJo 9.6.6 and FlowJo 10.6.1 (FlowJo) - used for flow cytometric data analysis
4) Prism 7 (GraphPad Software) - used to generate graphs and perform statistics
5) BZ-X Analyzer 1.4.0.1(Keyence), cellSens 1.16 (Olympus), ZEN software 2.3 SP1 FP_3 (Zeiss) and NIS-Elements 4.13.05 (Nikon)- used to anlyze images of mouse tissues
6) R 4.0.2 and R 3.5.1 software - used for analyzing gene expression and disease-free survival information. Statical analyses were conducted with following packages: MASS 7.3.51.4, stats 3.6.1, ggplot2 3.2.1 and BiocManager 1.30.10.
In RNA-seq analysis, adapter trimming and quality filtering of resulting fastq files were performed by using Cutadapt-v1.18. Trimmed reads were mapped to the Refseq mm10 RNA using Bowtie2-2.2.5. The sample to sample normalization was performed with TCC package. Normalized data were then tested for differential gene expression using the TCC package, which integrates the edgeR package.
7) DAVID Bioinformatics Resources (https://david.ncifcrf.gov/home.jsp) - used for GO enrichment analysis of RNAseq data
8) Agilent Feature Extraction Software (v11.0.1.1) and Gene Ontology (http://geneontology.org) - used for microarray analysis
9) Gene Set Enrichment Analysis (GSEA) software (version 4.1, Broad Institute) - used to GSEA analysis
10) Fastp 0.20.1, Salmon 0.8.0, and DEseq2 1.14.1 -these packages were used to analysis for the data set E-MTAB-7765

For manuscripts utilizing custom algorithms or software that are central to the research but not yet described in published literature, software must be made available to editors/reviewers. We strongly encourage code deposition in a community repository (e.g. GitHub). See the Nature Research guidelines for submitting code & software for further information.

Data

Policy information about availability of data

All manuscripts must include a data availability statement. This statement should provide the following information, where applicable:

- Accession codes, unique identifiers, or web links for publicly available datasets
- A list of figures that have associated raw data
- A description of any restrictions on data availability

Microarray and RNA-seq data have been deposited in the GEO repository under the accession code GSE141643 and GSE164232. GSE141644 is the reference series of this publication.
Other datasets referenced during the study are available from the GEO repository under the accession number GSE33113, GSE35602, GSE17536 and GSE17537, and from ArrayExpress database under accession code E-MTAB-7764.
Referenced manuscripts and links of the datasets are listed below.
GSE33113 (Cell Stem Cell 2011 Nov 4;9(5):476-85. PMID: 22056143, https://www.ncbi.nlm.nih.gov/geo/query/acc.cgi?acc=GSE33113)
GSE35602 (Clin Cancer Res 2012 Jun 1;18(11):3054-70. PMID: 22452939, https://www.ncbi.nlm.nih.gov/geo/query/acc.cgi?acc=GSE35602)
GSE17536, GSE17537 (Gastroenterology 2010 Mar;138(3):958-68. PMID: 19914252, GSE17536: https://www.ncbi.nlm.nih.gov/geo/query/acc.cgi?acc=GSE17536, GSE17537: https://www.ncbi.nlm.nih.gov/geo/query/acc.cgi?acc=GSE17537)
GSE14333 (Clin Cancer Res 2009 Dec 15;15(24):7642-7651. PMID: 19996206: https://www.ncbi.nlm.nih.gov/geo/query/acc.cgi?acc=GSE14333)
E-MTAB-7764 (Gut 2020 Jul;69(7):1269-1282. PMID: 31685519, https://www.ebi.ac.uk/arrayexpress/experiments/E-MTAB-7764/)

Source data is provided with this paper as Excel file and includes Figures 1c, 1d, 1e, 1f, 1i, 1l, 2a, 2c, 2d, 2g, 2i, 2j, 3a, 3e, 3f, 3i, 4a, 4c, 4e, 5a, 5b, 5c, 5d, 5f, 5g, 5i, 6b, 6c, 6d, 6f, 7b, 7c, 7d, 7f, 7h, 7i, 8a, 8b, 8c, 8d, 8f, 9b, 9c, 10a and Supplementary Figures 1b, 1c, 1h, 2a, 2b, 2c, 2d, 3b, 4e, 5a, 6a, 6b, 6c, 7a, 7c, 7d, 7f, 8, 9a and 10a.
All the other relevant data are available from the corresponding authors upon reasonable request.

Field-specific reporting

Please select the one below that is the best fit for your research. If you are not sure, read the appropriate sections before making your selection.

☒ Life sciences ☐ Behavioural & social sciences ☐ Ecological, evolutionary & environmental sciences

For a reference copy of the document with all sections, see nature.com/documents/nr-reporting-summary-flat.pdf

Life sciences study design

All studies must disclose on these points even when the disclosure is negative.

Sample size	We did not performed sample-size calculation. Sample size was determined to be adequate based on the magnitude and consistency of measurable differences between groups. Sample sizes for each experiment was described in the manuscript. Ultimately at least two independent experiments were performed to draw conclusions.
Data exclusions	No data was excluded in all our experiments.
Replication	We confirmed that all attempts at replication were successful. Ultimately at least two independent experiments were performed to draw conclusions.
Randomization	In mouse experiments, no randomization was performed. Animals were compared with cohoused and/or cohoused control litter-mate controls. For in vitro experiments, control and experimental groups are always randomly assigned.
Blinding	During assessment of tumor size, assessors did not know the genotypes of the mice. No blinding was used in vitro experiments, flow cytometry and immunohistochemical analysis. The analysis of these data was strictly quantitative and not subjective and the data collection analysis were performed with quantitative instruments to maintain objectively.

Reporting for specific materials, systems and methods

We require information from authors about some types of materials, experimental systems and methods used in many studies. Here, indicate whether each material, system or method listed is relevant to your study. If you are not sure if a list item applies to your research, read the appropriate section before selecting a response.

Materials & experimental systems

n/a	Involved in the study
☐	☒ Antibodies
☐	☒ Eukaryotic cell lines
☒	☐ Palaeontology
☐	☒ Animals and other organisms
☐	☒ Human research participants
☒	☐ Clinical data

Methods

n/a	Involved in the study
☒	☐ ChIP-seq
☐	☒ Flow cytometry
☒	☐ MRI-based neuroimaging

Antibodies

Antibodies used

Immunohistochemistry:
anti-phospho-ERK (4370, Lot. 17, CST, 1:800)
anti-Ki67 (ab16667, Abcam, 1:100)
anti-GFP (GFP-Go-Af1480 or GFP-Rb-Af2020, Frontier Institute, 1:200)
anti-BrdU (BU1/75, BIO-RAD, 1:100)
anti-human IL-11 (LS-C408373, Lot. 132036, LSBio. 1:500)
anti-mouse IL-11 (clone #12, in house, 750ng/mL)
anti-CD45 (13917, Lot. 1, CST, 1:200)
anti-podoplanin (127404, Lot. B205843, BioLegend, 1:100)
anti-α-SMA (ab5694, Lot. GR283004-32, Abcam, 1:200)
anti-collagen I (ab34710, Lot. GR306872-1, Abcam, 1:500)
anti-collagen IV (ab6586, Lot. GR292187-3, Abcam, 1:500)
anti-E-cadherin (560062, Lot. 4269828, BD Biosciences, 1:200)
anti-vimentin (9856, Lot. 8, CST, 1:200)
anti-cleaved caspase-3 (Asp175) (9664, CST, 1:2000)
anti-phospho-ERK (4370, CST, 1:800)
anti-Wnt5a/b (C27E8, CST, 1:50)
anti-Ki67 (ab16667, Abcam, 1:100)
Alexa Fluor 488-conjugated donkey anti-rabbit IgG (A21206, Lot. 1531671, Invitrogen, 1:500)
Alexa Fluor 594-conjugated donkey anti-rabbit IgG (A21207, Lot. 1744751, Invitrogen, 1:500)
Alexa Fluor Plus 594-conjugated donkey anti-rabbit IgG (A32754, Lot. TI271728, Invitrogen, 1:500)
Alexa Fluor 647-conjugated donkey anti-rabbit IgG (A31573, Lot. 1964354, Invitrogen, 1:500)
Alexa Fluor 594-conjugated donkey anti-mouse IgG (A21203, Lot. 1820027, Invitrogen, 1:500)
Alexa Fluor 488-conjugated donkey anti-goat IgG (A11055, Lot. 1915848, Invitrogen, 1:500)
Streptavidin, Alexa Fluor 594-conjugate (S11227, Invitrogen, 1:500)
ImmPRESS® VR anti-rabbit IgG HRP polymer detection kit (MP-6401, Lot. ZF0725, Vector Laboratories, 1:1)
Biotinylated Goat anti-Rabbit Immunoglobulins (E0432, Lot. 20050577, Dako, 1:200)

Flow cytometry:
anti-CD11b (20-0112, clone M1/70, Lot. C0112092313203, TONBO, 1:100)
anti-CD16/CD32-mAb (2.4G2) (made in house, 0.01 mg/mL)
anti-CD24a (101813, clone M1/69, Lot. B161564, Biolegend, 1:100)
anti-CD29 (102207, BioLegend, HM-beta 1-1, 1:100)
anti-CD31 (17-0311-82, clone 390, Lot. 4330203, eBioscience, 1:250)
anti-CD34 (13-0341-81, clone RAM34, Lot. E02496-1630, eBioscience, 1:100)
anti-CD45.1 (35-0453, clone A20, Lot. C0453030713353, TONBO, 1:100)
anti-CD45.2 (20-0454,clone 104, Lot. C0454010713203, TONBO, 1:100)
anti-EpCAM (118214, clone G8.8, Lot. B173068, BioLegend, 1:250)
anti-Thy1.2 (20-0903,clone 30-H12, Lot. C0903041913203, TONBO, 1:250)
anti-podoplanin (127410, clone 8.1.1, Biolegend, 1:250)
anti-TER-119 (116212, clone TER-119, BioLegend, 1:100)
anti-MHC Class II (130-102-139, clone M5/114.15.2, Miltenyi Biotec, 1:250)
anti-ICAM-1 (561605, clone 3E2, BD Biosciences, 1:100)
anti-VCAM-1 (105718, clone 429, Lot. B179439, BioLegend, 1:100)
anti-Sca-1 (122512, clone E13161.7, Lot. B177105, BioLegend, 1:250)
anti-Lyve1 (50-0443-80, clone ALY7, Lot. E12123-1632, eBioscience, 1:100)
anti-PDGFRα (17-1401-81, clone APA5, Lot. E07220-1631, eBioscience, 1:100)

Western Blotting:
anti-phospho-STAT3 (9145, Lot. 34, CST, 1:1000)
anti-STAT3 (SC-482, Lot. J2108, Santa Cruz, 1:1000)
anti-phospho-ERK (4370, CST, 1:1000)
anti-ERK (9102, CST, 1:1000)
anti-β-Actin (622102, Lot. B169853, Biolegend, 1:1000)
anti-tubulin (T5168, Lot. 42K4827, Sigma-Aldrich, 1:50000)
Horseradish peroxidase (HRP) -conjugated anti-rabbit IgG (NA934, Lot. 16959202, GE Healthcare, 1:5000)
HRP-conjugated anti-mouse IgG (NA931, Lot. 14890050, GE Healthcare, 1:5000)

	Neutralizing antibody:<br>anti-TGF-beta antibody (clone 1D11, in-house, 5 mg/kg)<br>Control mouse IgG (I5381, Sigma-Aldrich, 5 mg/kg)
Validation	All antibodies that came from commercial vendors are validated by the manufactures for the species and assay in our study. Validation data is available on the manufacture's websites.<br>All antibodies were initially tested against unstained controls and dilution series were performed to optimize.<br>About anti-mouse IL-11 (clone #12, in-house), this was validated in our previous publication (Deguchi et al., BBRC, 2018, PMID: 30268501).<br>About anti-TGF-beta antibody (clone 1D11, in-house), this was validated in (Dasch et al., J Immunol, 1989, PMID: 2537357)

Eukaryotic cell lines

Policy information about cell lines

Cell line source(s)	A human breast cancer cell line, MDA-MB-231 was provided by Dr. T Sakamoto (Kanazawa University), which was obtained from the American Type Culture Collection (ATCC).<br>Human colon cancer cell lines, HT29, and HCT116 cells were obtained from ATCC.<br>The hybridoma cell lines, 1D11, and 2.4G2 were obtained from ATCC.<br>HEK293 cells stably expressing mouse Wnt3a and human afamin was provided by J. Takagi (Osaka University).<br>Cultrex® R-spondin1 (Rspo1) Cells were obtained from Trevigen.
Authentication	HT29, HCT116, and MDA-MB-231 cells were not authenticated by STR analysis after obtaining from ATCC, but their morphologies and reactivities to species-specific antibodies were consistent with their designated origins.<br><br>1D11 and 2.4G2 hybridoma were authenticated by analyzing the production of their specific antibodies.<br><br>Characterization of HEK293 cells stably expressing Afamin and Wnt3a was previously published (Mihara et al., eLife, 2016).<br><br>Cultrex® R-spondin1 (Rspo1) Cells were authenticated by Trevigen.<br>The production of R-spondin1 were observed in the cells as their designated origins.<br><br>All cell lines were kept at low passages in order to maintain their identity.
Mycoplasma contamination	The cell lines were not tested for mycoplasma contamination.
Commonly misidentified lines<br>(See ICLAC register)	None of misidentified cell lines were used this study.

Animals and other organisms

Policy information about studies involving animals; ARRIVE guidelines recommended for reporting animal research

Laboratory animals	All mouse strains are described in the material and methods section, both female and male mice were involved. The age of the mice used in each experiment is either stated in the methods section and/or in the figure legends for each experiment.<br><br>The mice were housed at 23 ± 2°C, a humidity of 55 ± 5%, and a 12 hr dark/light cycle.
Wild animals	This study did not involve wild animals.
Field-collected samples	This study did not involve samples collected from the field.
Ethics oversight	All experiments were performed according to the guidelines approved by the Institutional Animal Experiment Committee of Juntendo University School of Medicine or Toho University School of Medicine (19-51-414 and 19-51-411).

Note that full information on the approval of the study protocol must also be provided in the manuscript.

Human research participants

Policy information about studies involving human research participants

Population characteristics	Human colon tumors and adjacent normal tissues from surgical section at Toho University Omori Medical Center (Tokyo, Japan) were collected between January 2006 and December 2016.<br>The samples were obtained from both men and females, and the ages of patients were between 48 and 81.
Recruitment	The tumor samples were primary tumors. Colon tumors included 11 cases of adenomas, 10 cases of early cancers, and 10 cases of advanced cancers. Histological assessment of adenomas and adenocarcinomas was according to the guidelines of the World Health Organization. According to the TNM criteria (pT1, early cancer; pT2-4, advanced cancers), we divided colon cancers into early and advanced colon cancers, respectively.<br><br>This study is retrospective study. We can not obtain individual consent from all patients. The presented study design was accepted by the ethics committee on the condition that a document that declares an opt-out policy by which any possible

	patients and relatives could refuse to be included in this study was uploaded on the Web page on the Toho University https://www.toho-u.ac.jp/med/med_research_index/clinical_specimen/kentai_a16111.html.
Ethics oversight	Human colon tumors and adjacent normal tissues were analyzed in accordance with approval by the Ethics Committee of Toho University School of Medicine (A16111).

Note that full information on the approval of the study protocol must also be provided in the manuscript.

Flow Cytometry

Plots

Confirm that:

☐ The axis labels state the marker and fluorochrome used (e.g. CD4-FITC).

☐ The axis scales are clearly visible. Include numbers along axes only for bottom left plot of group (a 'group' is an analysis of identical markers).

☒ All plots are contour plots with outliers or pseudocolor plots.

☒ A numerical value for number of cells or percentage (with statistics) is provided.

Methodology

Sample preparation	To isolate IL-11+ (EGFP+) cells, the colon or tumors were removed from DSS-treated or AOM/DSS-treated Il11-Egfp reporter mice, or APCmin/+; Il11-Egfp reporter mice. Then, tissue samples were minced with scissors and digested in RPMI 1640 containing 1 mg/mL Collagenase (Wako), 50 µg/mL DNase (Roche), 0.5 mg/mL Dispase (Roche), 100 U/mL Penicillin, and 100 µg/mL Streptomycin, and 2% (v/v) fetal bovine serum (FBS, Gibco) for 60 min. Single cell suspensions were prepared, and cells were stained with the indicated antibodies and analyzed.<br>In cell sorting, tissue samples were minced with scissors and digested in RPMI 1640 containing 0.25 mg/mL Liberase (Sigma), 50 µg/mL DNase (Roche), 100 U/mL Penicillin, and 100 µg/mL Streptomycin, and 5% (v/v) fetal bovine serum (FBS, Gibco) for 60 min. EGFP+ and EGFP- cells among Ter119- CD45- CD31- EpCAM- cell populations were enriched using MojoSort Mouse anti-APC Nanobeads (BioLegend) and MojoSort Magnet (BioLegend).
Instrument	LSRFortessa X-20 (BD Biosciences)<br>BD Verse (BD Biosciences)<br>BD FACSAria III Cell Sorter (BD Biosciences)
Software	BD FACSDiva Software Version 8.0.1 (BD Bioscience)<br>BD FACSuite Software (BD Bioscience)<br>FlowJo software Version 10.7.1 (FlowJo)
Cell population abundance	In lineage marker expression analysis, at least 100,000 live cells were analyzed.<br>In cell sorting, at least 1,0,000 live cells were obtained.
Gating strategy	For lineage marker expression analysis,<br>(1) Cells were identified based on FSC-A/SSC-A.<br>(2) GFP positive cells were identified by FSC-A/EGFP-A.<br>(3) Doublets were excluded based on FSC-A/FSC-H, FSC-W/FSC-H, SSC-A/SSC-H and SSC-W/SSC-H.<br>(4) Live cells were selected by gating on DAPI negative events.<br>(5) Lineage marker expressions were analyzed in EGFP-positive events.<br><br>For cell sorting,<br>(1) Cells were identified based on FSC-A/SSC-A.<br>(2) Doublets were excluded based on FSC-A/FSC-H, FSC-W/FSC-H, SSC-A/SSC-H and SSC-W/SSC-H.<br>(3) Live cells were selected by gating on DAPI negative events.<br>(4) GFP positive cells were identified by SSC-A/EGFP-A.<br>(5) GFP positive and negative stromal cells were selected by gating on APC-A negative events.

☒ Tick this box to confirm that a figure exemplifying the gating strategy is provided in the Supplementary Information.
